# Histone Deacetylase Inhibitor Romidepsin Induces HIV Expression in CD4 T Cells from Patients on Suppressive Antiretroviral Therapy at Concentrations Achieved by Clinical Dosing

**DOI:** 10.1371/journal.ppat.1004071

**Published:** 2014-04-10

**Authors:** Datsen George Wei, Vicki Chiang, Elizabeth Fyne, Mini Balakrishnan, Tiffany Barnes, Michael Graupe, Joseph Hesselgesser, Alivelu Irrinki, Jeffrey P. Murry, George Stepan, Kirsten M. Stray, Angela Tsai, Helen Yu, Jonathan Spindler, Mary Kearney, Celsa A. Spina, Deborah McMahon, Jacob Lalezari, Derek Sloan, John Mellors, Romas Geleziunas, Tomas Cihlar

**Affiliations:** 1 Gilead Sciences, Foster City, California, United States of America; 2 Division of Infectious Diseases, Department of Medicine, University of Pittsburgh School of Medicine, Pittsburgh, Pennsylvania, United States of America; 3 HIV Drug Resistance Program, Center for Cancer Research, National Cancer Institute, Frederick, Maryland, United States of America; 4 Department of Pathology, School of Medicine, University of California San Diego, La Jolla, California, United States of America; 5 Quest Clinical Research, San Francisco, California, United States of America; Miller School of Medicine, United States of America

## Abstract

Persistent latent reservoir of replication-competent proviruses in memory CD4 T cells is a major obstacle to curing HIV infection. Pharmacological activation of HIV expression in latently infected cells is being explored as one of the strategies to deplete the latent HIV reservoir. In this study, we characterized the ability of romidepsin (RMD), a histone deacetylase inhibitor approved for the treatment of T-cell lymphomas, to activate the expression of latent HIV. In an in vitro T-cell model of HIV latency, RMD was the most potent inducer of HIV (EC_50_ = 4.5 nM) compared with vorinostat (VOR; EC_50_ = 3,950 nM) and other histone deacetylase (HDAC) inhibitors in clinical development including panobinostat (PNB; EC_50_ = 10 nM). The HIV induction potencies of RMD, VOR, and PNB paralleled their inhibitory activities against multiple human HDAC isoenzymes. In both resting and memory CD4 T cells isolated from HIV-infected patients on suppressive combination antiretroviral therapy (cART), a 4-hour exposure to 40 nM RMD induced a mean 6-fold increase in intracellular HIV RNA levels, whereas a 24-hour treatment with 1 µM VOR resulted in 2- to 3-fold increases. RMD-induced intracellular HIV RNA expression persisted for 48 hours and correlated with sustained inhibition of cell-associated HDAC activity. By comparison, the induction of HIV RNA by VOR and PNB was transient and diminished after 24 hours. RMD also increased levels of extracellular HIV RNA and virions from both memory and resting CD4 T-cell cultures. The activation of HIV expression was observed at RMD concentrations below the drug plasma levels achieved by doses used in patients treated for T-cell lymphomas. In conclusion, RMD induces HIV expression ex vivo at concentrations that can be achieved clinically, indicating that the drug may reactivate latent HIV in patients on suppressive cART.

## Author Summary

Combination antiretroviral therapy has greatly improved the clinical outcome of HIV infection treatment. However, latent viral reservoirs established primarily in memory CD4 T cells persist even after long periods of suppressive antiretroviral therapy, which hinders the ability to achieve a prolonged drug-free remission or a cure of the HIV infection. Activation of HIV expression from latent reservoirs is a part of proposed strategies that may potentially lead to virus elimination and ultimately cure of the infection. In this study, we show that romidepsin, a histone deacetylase inhibitor approved for the treatment of T-cell lymphomas, is a potent activator of HIV expression in an in vitro model of viral latency as well as ex vivo in resting and memory CD4 T cells isolated from HIV-infected patients with suppressed viremia. Importantly, the ex vivo activation of latent HIV occurred at romidepsin concentrations lower than those achieved in drug-treated lymphoma patients. In addition, romidepsin exhibited a more potent effect than other drugs in the same class that have already been shown to activate HIV expression in vivo. Together, these results support the clinical assessment of romidepsin in HIV-infected patients on suppressive antiretroviral therapy.

## Introduction

Combination antiretroviral therapy (cART) has dramatically improved the life expectancy and health of patients infected with HIV. In the setting of controlled clinical trials with optimal cART, up to 90% of treatment-naïve patients can achieve undetectable virus in plasma and normalization of CD4 T-cell levels [1], [2]. However, when cART is interrupted in patients who initiated therapy during the chronic phase of infection, virus replication resumes in virtually all patients [3]–[5], indicating that current cART is not sufficient to cure HIV infection. The failure of cART to cure HIV infection is due, in part, to the ability of HIV to establish latency in a subset of infected CD4 T cells [6]. The state of latency is characterized by the presence of integrated but transcriptionally silent proviral HIV DNA, which makes the infected cells invisible to the immune system and resistant to both innate antiviral defenses and antiretroviral therapy [6], [7].

Although latent proviral DNA has been detected in multiple different immune cell subsets permissive to HIV infection, long-lived resting memory CD4 T cells are believed to represent the predominant reservoir of proviruses that can be activated to produce infectious virions [8], [9]. Initial quantification of latent HIV proviruses in peripheral blood lymphocytes from patients on cART revealed approximately 200 copies per 10^6^ resting CD4 T cells; however, in general, less than 1% of these proviruses was shown to produce infectious HIV after T-cell mitogenic stimulation with substantial inter-patient variation observed in the fraction of total proviruses that could be activated [10]. The pool of latently infected memory CD4 T cells is believed to be maintained throughout a patient's life by homeostatic proliferation of memory T cells and/or intermittent antigen-driven clonal expansion [11]. Alternatively, low levels of HIV replication confined to lymphatic tissues and undetectable in the periphery may also contribute to the maintenance of the latent virus reservoir [11], [12]. The decay rate of latent virus reservoirs in peripheral blood lymphocytes has been estimated to have a half-life of >3 years, indicating that even life-long cART is unlikely to cure HIV infection [7].

Chronic HIV infection, even when suppressed by cART, poses long-term health risks that include accelerated cardiovascular disease, liver and renal disease, non-AIDS-associated cancers, neurocognitive impairment, and accelerated senescence of immune responses [13]–[15]. Thus, there is a clear unmet medical need for novel therapeutic interventions that could lead either to host-mediated control of HIV in the absence of cART or complete clearance of viral reservoirs. Such virus eradication interventions will need to be well tolerated with minimal side effects. Specifically, therapeutic interventions need to be found that do not cause global T cell activation or a chronic state of inflammation. These interventions should also exhibit minimal drug-drug interactions with medications frequently administered to HIV-infected patients.

Identifying a safe modality to activate latent HIV in memory CD4 T cells is an important goal and potentially represents the first key step towards a cure for HIV. Establishment and maintenance of HIV latency is a complex process that appears to involve multiple mechanisms restricting productive viral transcription. These mechanisms include promoter occlusion via steric hindrance, insufficient levels of cellular transcription factors, modification of the HIV 5′ long-terminal repeat (LTR) by methylation and alteration of the chromatin environment in the vicinity of the LTR by histone deacetylation and other epigenetic modifications [16], [17]. Histone deacetylases (HDACs) have been implicated in maintaining HIV in a latent state. In this process, HDACs are recruited to the LTR by various transcriptional regulators and deacetylate lysine residues on histones, inducing chromatin condensation, thereby repressing proviral transcription [18], [19]. Consistent with this mechanism, HDAC inhibitors (HDACi) have been reported to activate latent HIV in cell lines and primary cells, including CD4 T cells from HIV-infected patients on cART [20], [21]. Vorinostat (suberoylanilide hydroxamic acid; VOR) is an HDACi approved for clinical use to treat cutaneous T-cell lymphomas and has also been shown to activate HIV transcription in various latency models [22]–[24]. Administration of a single dose of VOR to eight HIV-infected patients on cART increased HIV RNA levels in resting CD4 T-cells by a mean of 4.8-fold [25]. Although it is unclear whether a single dose of VOR diminished the activatable latent HIV reservoir, these results represent an important milestone by demonstrating that HIV expression can be increased pharmacologically in HIV-infected patients on cART.

In a recent study exploring a panel of HDACi for HIV activation using an in vitro latency assay, panobinostat (LBH589; PNB) displayed superior potency to multiple other HDACi tested including givinostat, belinostat, and VOR [26]. Notably, a potent HDACi romidepsin (RMD; Istodax) was not tested in this study. RMD is a cyclic peptide naturally synthesized by *Chromobacterium violaceum* [27] that has received regulatory approval for the treatment of patients with peripheral T-cell lymphomas (PTCL) or cutaneous T-cell lymphoma (CTCL) [28].

The present study explores the ability of RMD, in comparison with VOR and other HDACi currently in clinical development, to reverse HIV latency in vitro in primary T cells infected with a reporter virus as well as ex vivo in resting and memory CD4 T cells from HIV-infected patients on suppressive cART.

## Results

### Romidepsin is a potent activator of HIV in an in vitro model of latency

To assess clinically tested HDACi for their ability to activate HIV from latency, we first employed a previously described in vitro HIV latency model [29], [30] with several modifications to increase the sensitivity of detection. The assay involves freshly isolated naïve CD4 T cells from healthy donors polarized to a Th0 phenotype, which mimics memory CD4 T cells. These Th0 cells are then infected with HIV expressing a luciferase reporter gene and cultured for an additional 7 to 10 days until a latent infection is established.

**Figure 1:**
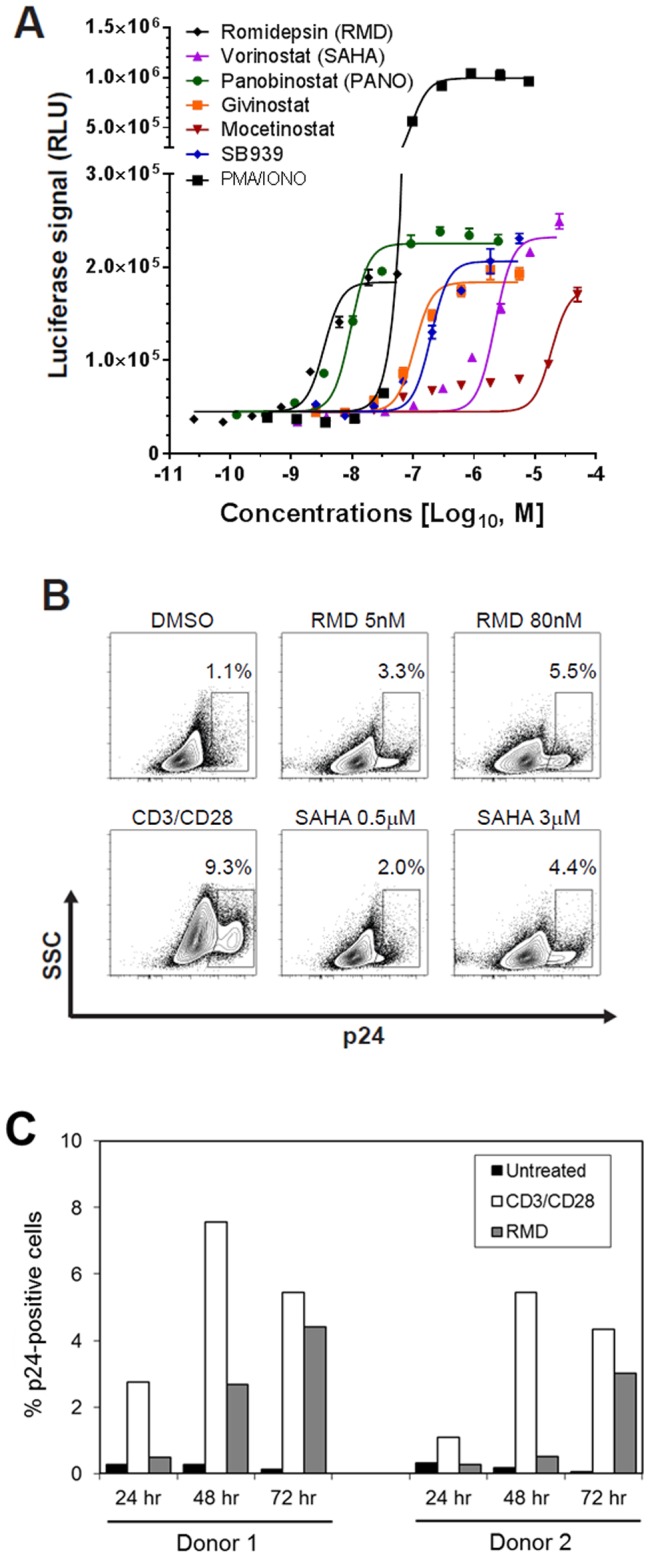
In vitro activation of HIV expression by HDAC inhibitors in an in vitro latency model. Primary CD4 T cells latently infected in vitro with reporter HIV were established as previously described [29], [30] with additional minor modifications described in Materials and Methods. The infected cells were incubated in the presence of the indicated HDACi. (**A**) A dose response of HIV activation by HDACi was determined by the quantification of luciferase reporter activity after a 48-hour treatment. Results are mean ± SD from a representative experiment performed in quadruplicate. (**B**) Induction of p24 expression by RMD and VOR. Flow cytometry analysis of cells from a representative donor is shown with gating on the live cell population. Anti-CD3/CD28 antibodies conjugated to beads were used as a positive control. (**C**) Time course of the induction of p24 expression by RMD. Cells isolated from 2 independent donors were treated with 40 nM RMD or anti-CD3/CD28 antibodies for 24 to 72 hours in the presence of antiretrovirals. Percentage of p24-positive cells was determined by flow cytometry with gating on live cell population.

The HDACi that we tested in this in vitro HIV latency assay included RMD, VOR, PNB, givinostat, mocetinostat, and pracinostat (SB939). All compounds showed dose-dependent activity in the assay, but displayed varying levels of potency in activating HIV expression (Fig. 1A). Based on experiments conducted with cells prepared from three independent donors, RMD was the most potent HDACi with a mean EC_50_ value of 4.5 nM (Table 1). Global T-cell activators such as anti-CD3/CD28 antibodies and PMA+ ionomycin consistently showed 2- to 4-fold higher maximum induction of the luciferase signal relative to RMD (data not shown). When cytotoxicity was determined under the identical conditions, RMD displayed a CC_50_ (50% cell viability reduction) value of 100 nM, resulting in an approximately 20-fold selectivity window (Table 1). PNB was the second most potent compound tested with an EC_50_ value of 10 nM and a relatively high selectivity window of >250-fold. VOR was substantially less potent in this assay with EC_50_ and CC_50_ values of 4 µM and >25 µM, respectively.

**Table 1:** Activity and cytotoxicity of HDAC inhibitors in the in vitro HIV latency model.

	EC_50_ (nM)	CC_50_ (nM)
RMD	4.5±1.0	107±126
VOR	3,950±1,900	>25,000
PNB	10.1±1.0	>2,500
Givinostat	95.8±20.3	24,000±5,020
Mocetinostat	13,600±4,664	10,100±106
SB939	212±14	>50,000

Latently infected primary CD4 T cells prepared from three independent healthy donors were treated with the indicated compounds continuously for 48 hours. EC_50_ and CC_50_ values were determined from each dose-dependent response using a multi-parameter algorithm. Data are mean ± SD from three independent experiments performed in quadruplicate.

To confirm that the HDACi were activating HIV expression in this latency model, we used flow cytometry analysis to quantify intracellular p24 antigen levels following treatment of latently infected CD4 T-cell cultures with RMD, VOR, and the positive control anti-CD3/CD28 antibodies (Fig. 1B). Treatment with 5 and 80 nM RMD, representing the minimal and maximal concentrations leading to the activation of HIV expression in this model, resulted in 3.3% and 5.5% of cells expressing p24 antigen, respectively. In comparison, treatment with 3.0 µM VOR induced p24 antigen expression in approximately 4.4% of cells. As expected, anti-CD3/CD28 antibodies induced p24 antigen expression in 2- to 3-fold higher fractions of CD4 T cells than RMD (Fig. 1B). While the p24 expression in anti-CD3/CD28-treated cells reached a plateau at 48 hours, the fraction of p24 positive cells in RMD-treated cultures continued to increase for 72 hours (Fig. 1C). These data indicate that RMD induces the expression of HIV proteins following the activation of latent provirus in vitro.

### HIV activation by HDACi correlates with the inhibition of HDAC isoenzymes

Since the specific mechanism of HIV latency reversal by HDACi remains to be fully understood, we investigated whether the relative potency of selected compounds in the HIV latency assay correlates with their ability to directly inhibit the activity of individual HDAC isoenzymes. RMD, VOR, and PNB were tested against 11 individual HDAC isoenzymes (HDAC-1 to -11) from four distinct classes: 1, 2a, 2b, and 4. Overall, RMD was the most potent inhibitor, especially against class 1 HDACs (HDAC-1 to -3) as well as HDAC-10 and HDAC-11 isoenzymes (Table 2). PNB showed lower potency, particularly against class 1 and 4 enzymes with up to 40-fold higher IC_50_ values relative to RMD. VOR was a substantially weaker inhibitor of the majority of HDAC enzymes than RMD with the exception of HDAC-6. The greatest difference in potency between RMD and VOR was observed with the class 1 HDAC enzymes that were 60- to 2,500-fold more susceptible to RMD than VOR (Table 2). The greater activity of RMD against a broad range of HDAC enzymes correlated with its higher potency to activate HIV expression relative to VOR (∼1,000-fold difference; compare Tables 1 and 2).

**Table 2:** Effect of RMD, VOR, and PNB on the activity of individual human HDAC isoenzymes.

		IC_50_ (nM)		
HDAC Class	Enzyme	RMD	VOR	PNB
1	HDAC-1	0.102	148	4.35
	HDAC-2	0.376	418	5.28
	HDAC-3	0.211	509	6.04
	HDAC-8	24.6	1,700	31.9
2a	HDAC-4	314	27,000	442
	HDAC-5	1,160	20,800	336
	HDAC-7	3,405	101,550	10,825
	HDAC-9	8,910	31,650	4,805
2b	HDAC-6	488	27.1	5.74
	HDAC-10	0.133	527	2.54
4	HDAC-11	0.407	504	9.56

Recombinant human HDAC isoenzymes were incubated with the indicated HDACi and fluorogenic peptide substrates as described in Materials and Methods. IC_50_ values were calculated from 10-point dose-response curves using a multi-parameter curve fit. Data represent mean values from two independent experiments.

### RMD activates latent HIV ex vivo in CD4 T cells from virally suppressed patients

**Figure 2:**
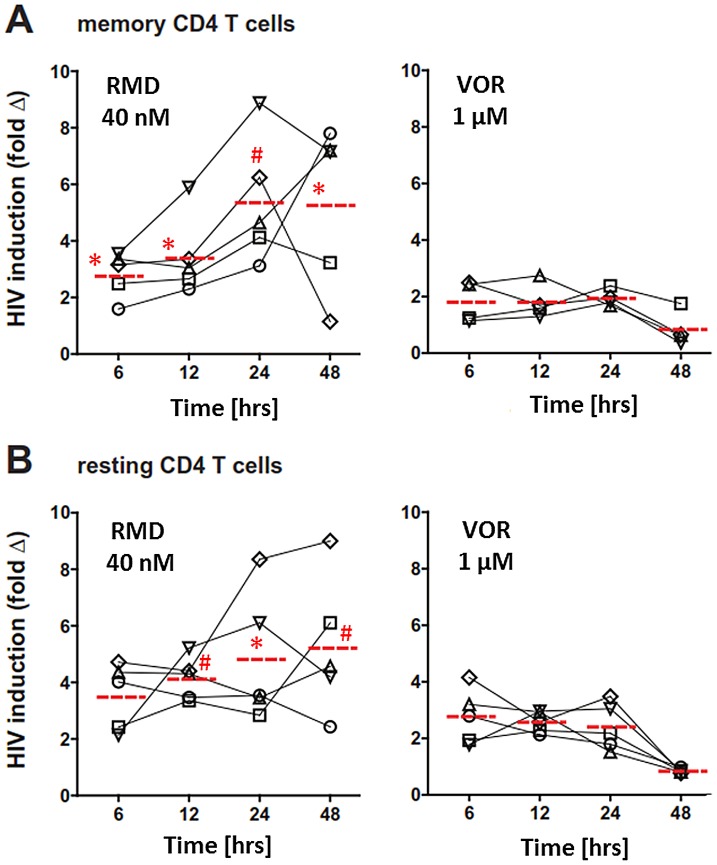
Ex vivo activation of HIV expression by RMD and VOR. CD4 T cells were isolated from virally suppressed HIV-infected patients and pulse-treated with RMD and VOR for 6 and 24 hours, respectively. Cell-associated total RNA was extracted, HIV RNA levels were quantified at the indicated time points, and fold increase in the cell-associated HIV RNA was determined relative to corresponding vehicle-treated control for each individual time point. The fold change for each donor and condition is based on mean number of HIV copies from 4 to 5 independent measurements. Red dashed line represents the mean fold HIV induction across all tested donors. Symbols # (p<0.01) and * (p<0.05) denote a statistically significant difference between fold HIV induction by RMD and VOR across all donors tested. Data for each individual donor and condition including results of statistical analysis are provided in Supplementary Table S4. (**A**) Memory CD4 T cells were purified as the CD4(+)CD45RA(−) subset. (**B**) Resting CD4+ T cells were purified as the CD4(+)HLA-DR(−)CD69(−)CD25(−) subset.

Previous studies revealed that VOR activates HIV transcription ex vivo in resting CD4 T cells isolated from HIV-infected patients on cART [21]. We used a similar approach to compare RMD to VOR in total memory as well as resting CD4 T cells isolated from patients chronically infected with HIV who were treated with cART and maintained their plasma HIV RNA at <50 copies/ml for at least 12 months (See Supplementary Table S1 for patient descriptions). In the initial set of experiments, isolated CD4 cells were treated with 40 nM RMD for 4 hours or 1 µM VOR for up to 24 hours to mimic the clinical exposure profiles of these drugs. To examine the kinetics of HIV induction, we analyzed cell-associated viral RNA levels at 6, 12, 24, and 48 hours after treatment initiation. Consistent with the data of Archin et al. [21], a 2- to 4-fold increase in HIV RNA levels was observed both in memory and resting CD4 T cells after 6 hours of VOR treatment compared with control vehicle (DMSO)-treated cells (Fig. 2). However, HIV RNA in VOR-treated cells decreased by 48 hours to levels detected in vehicle-treated cells. In contrast, cell-associated HIV RNA continued to increase in both memory and resting CD4 T cells isolated from the same donors following their exposure to RMD (Fig. 2). Levels of intracellular HIV RNA in both cell types were 5- to 6-fold higher compared with vehicle-treated controls and peaked between 24 and 48 hours after the addition of RMD. These findings suggest that the activation of HIV transcription with RMD is more durable than with VOR.

### Ex vivo treatment with RMD induces a release of extracellular HIV RNA

**Figure 3:**
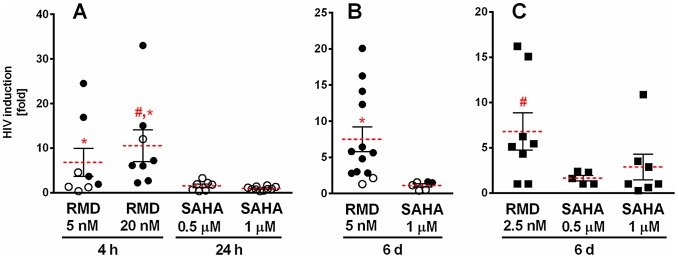
Induction of extracellular viral RNA release from CD4 T cells treated with RMD and VOR. Memory or resting CD4 T cells isolated from HIV-infected patients on suppressive cART were treated with RMD or VOR, and viral RNA was quantified in cell culture supernatants 6 days after the addition of drugs. Results are depicted as fold increase in viral RNA relative to control cultures. Each symbol represents one HIV subject. Solid circles, p<0.05; open circles, p>0.05 compared to vehicle-treated controls from the same donors; solid squares, p value not calculated. Red lines represent the mean fold HIV induction across all analyzed donors. Symbols # and * denote a statistically significant difference (p<0.05) for RMD-mediated HIV induction vs. 0.5 and 1 µM VOR, respectively, across all tested donors. Data for each individual donor and condition including results of the statistical analysis are summarized in Supplementary Table S4. (**A**) Memory CD4 T cells were treated with RMD and VOR for 4 and 24 hours, respectively. (**B**) Memory CD4 T cells were treated continuously for 6 days. (**C**) Resting CD4 T cells were treated continuously for 6 days.

We next examined whether RMD and VOR induce the release of HIV particles from patient-derived memory and resting CD4 T cells. We used HIV RNA in cell culture supernatants as a surrogate marker for virion release from treated cells. We assumed that extracellular virions would accumulate over the course of several days; therefore, the duration of culture was extended to 6 days to maximize assay sensitivity. Memory CD4 T cells from multiple patients were treated with 5 and 20 nM RMD for 4 hours, or 0.5 and 1 µM VOR for 24 hours. Under these conditions, RMD, but not VOR, increased extracellular HIV RNA in culture supernatants of memory CD4 cells isolated from multiple HIV-infected virally suppressed patients (Fig. 3A). The release of viral RNA from cells treated with RMD is unlikely to be due to cytotoxicity of the compound because the tested concentrations did not substantially affect cell viability (Supplementary Fig. S1). In addition, highly cytotoxic compounds such as blasticidine caused >80% cell death during the same incubation period, but did not lead to release of detectable levels of HIV RNA into cell culture supernatants (data not shown).

To determine whether the observed lack of HIV RNA release from cells treated with VOR was due to an insufficient duration of drug treatment, memory and resting CD4 T cells isolated from HIV-infected patients on cART were treated with 1 µM VOR for 6 days. Similar to the cultures treated with VOR for 24 hours, there was no significant increase in HIV RNA in culture supernatants following a 6-day VOR treatment of memory CD4 T cells isolated from two HIV-infected patients on suppressive cART. In contrast, continuous 6-day treatment with RMD resulted in the detection of extracellular HIV RNA in memory CD4 T-cell cultures from the majority of tested donors (Fig. 3B). Continuous treatment with RMD was conducted at a lower drug concentration (5 nM) to avoid cytotoxicity. Similarly, 2.5 nM RMD induced HIV RNA release in resting CD4 T-cell cultures from 6 of 8 tested donors. In comparison, treatment of resting CD4 T cells with 1 µM VOR resulted in a measurable increase of extracellular HIV RNA in 3 of 7 patient-derived cultures tested, but 0.5 µM VOR did not increase extracellular HIV RNA significantly above the levels of untreated controls (Fig. 3C). Importantly, HIV RNA released into cell culture supernatants of the resting CD4 T cells treated with RMD can be pelleted by high-speed centrifugation, supporting the conclusion that the extracellular HIV RNA is associated with virion particles (Supplementary Table S2).

### HIV activation correlates with the inhibition of HDAC activity in resting CD4 T cells

**Figure 4:**
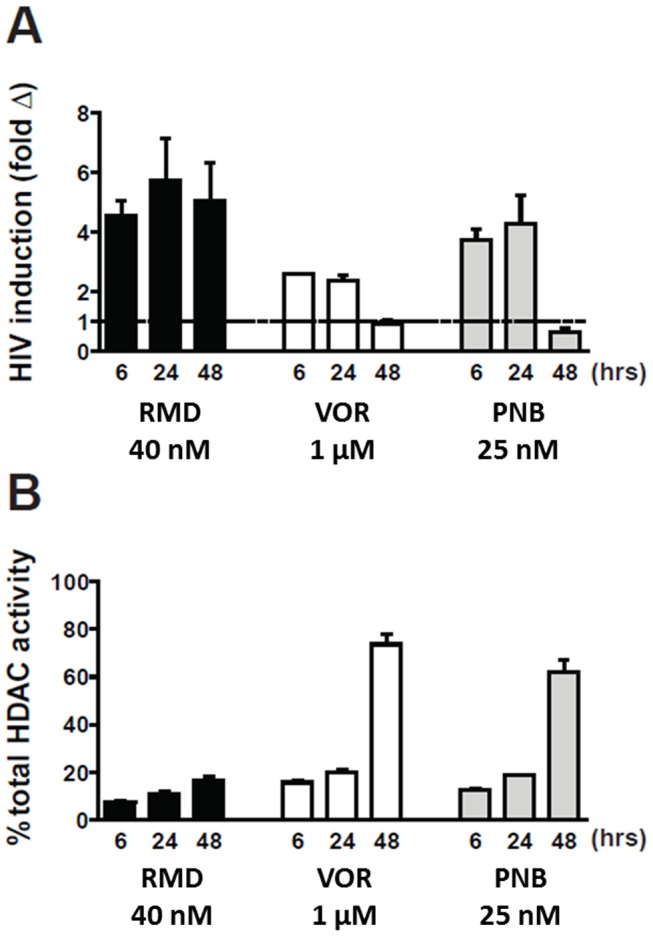
Induction of HIV expression and inhibition of cell-associated HDAC activity by HDACi. Resting CD4 T cells isolated from cART-suppressed HIV-infected patients were pulse-treated with RMD (4 hours), VOR (24 hours), and PNB (24 hours). Viral RNA was determined 48 hours after the initiation of the treatment. Data represent mean ± SD from two independent donors. (**A**) Cell-associated total RNA was extracted at the indicated time points, and HIV RNA levels were quantified. Data for each individual donor and condition including results of the statistical analysis are summarized in Supplementary Table S4. (**B**) Total class I and II HDAC enzyme activity was measured in total cell extracts of treated cells relative to vehicle-treated cells (representing 100% activity) using a model substrate described in Materials and Methods.

Although the relative in vitro HIV activation potency of RMD, VOR, and PNB correlated with the inhibitory activity of these compounds against multiple HDAC isoforms, we next set out to determine whether inhibition of cellular HDAC activity in latently infected cells also correlated with HIV activation. To address this question, resting CD4 T cells isolated from three cART-suppressed HIV-infected patients were treated with RMD, VOR, or PNB, and activation of intracellular HIV RNA expression was determined in parallel with cell-associated HDAC enzymatic activity. We found that the activation of HIV expression correlated with HDAC inhibition across multiple time points ranging from 6 to 48 hours (Fig. 4). In RMD-treated cells, both HIV activation and cell-associated HDAC inhibition persisted throughout the 48-hour incubation period. In contrast, the inhibition of HDAC activity by VOR and PNB was transient and decreased after 24 hours of incubation, which paralleled the levels of cell-associated HIV RNA over time (Fig. 4). Concentrations of VOR and PNB used in these experiments corresponded to 100% systemic drug exposure observed in treated cancer patients, while the concentration of RMD was 40% of the clinical drug exposure. These results reveal that sustained inhibition of cell-associated HDAC activity by RMD correlates with persistent activation of HIV expression.

### RMD reactivates latent HIV in resting CD4 T cells at concentrations substantially below those achieved during clinical dosing

**Figure 5:**
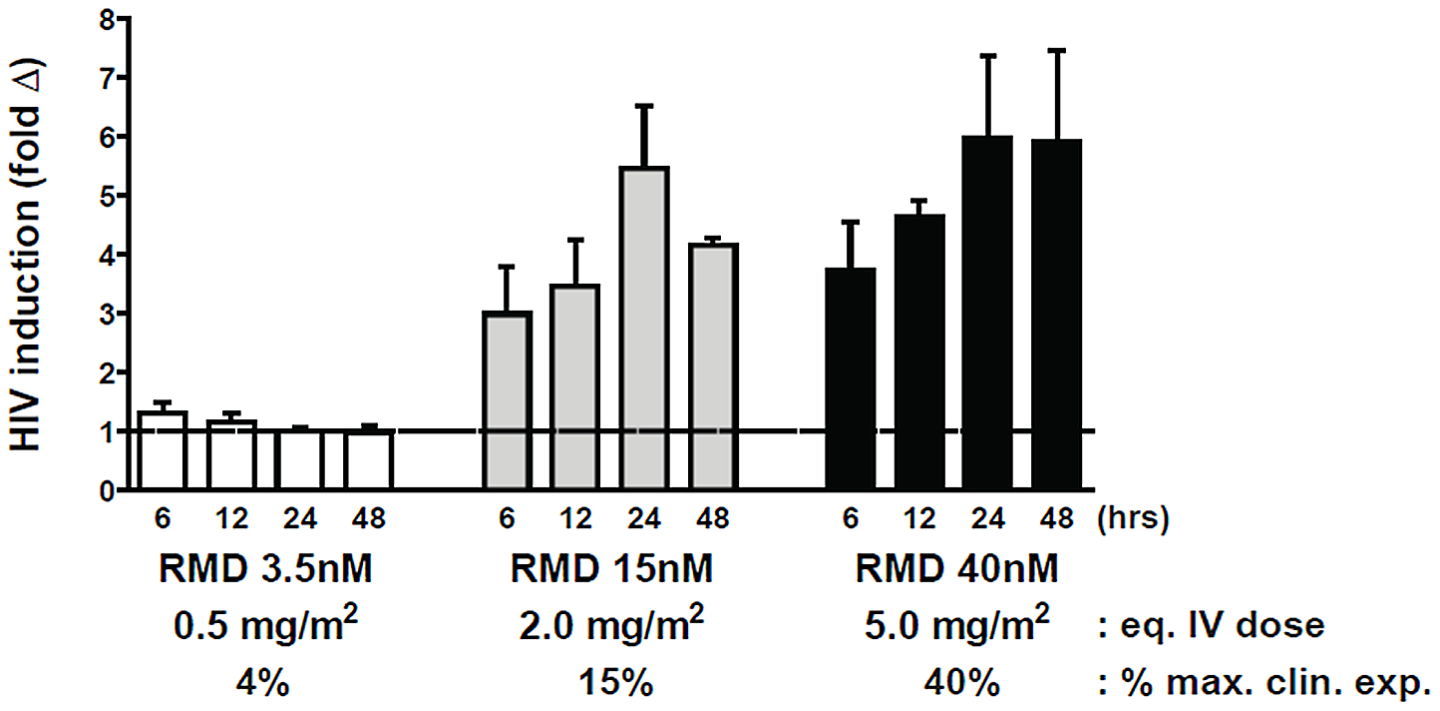
RMD activates intracellular HIV expression at concentrations below the levels achieved by clinical dosing. Resting CD4 T cells were isolated from 3 cART-suppressed HIV-infected patients and pulse-treated with RMD for 4 hours with the indicated concentrations. Cell-associated HIV RNA levels were analyzed at each time point following the treatment initiation (t = 0 hour), and fold induction was determined relative to a background signal in vehicle-treated controls. Predicted i.v. dose and percentage of clinical exposure were calculated for each RMD concentration tested relative to the clinically approved dose of 14 mg/m^2^; calculations were performed based on the free fraction of drug in human plasma and cell culture media. Data represent mean ± SD from at least 3 HIV-infected donors. Data for each individual donor and condition including results of the statistical analysis are summarized in Supplementary Table S4.

Since RMD-related toxicities have been observed at doses used for the clinical treatment of hematologic malignancies [31], [32], we tested whether RMD can activate latent HIV in patient-derived CD4 T cells at concentrations below its clinical exposure achieved with the dose of 14 mg/m^2^ used for the treatment of lymphomas [33]. Resting CD4 T cells from three HIV-infected patients were treated ex vivo with 3.5 to 40 nM RMD for 4 hours to mimic 40% or less of the systemic drug levels achieved during the i.v. administration of the clinical dose based on the relative free drug fraction in human plasma and cell culture media (Supplementary Table S3). While minimal induction of HIV transcription in resting CD4 T cells was observed with 3.5 nM RMD, treatment with 15 or 40 nM RMD induced 4- to 6-fold activation of HIV RNA expression (Fig. 5). This contrasted with results obtained following treatment with 1 µM VOR, which induced rapid, but transient and less-pronounced activation of latent HIV expression (Fig. 2 and Fig. 4A). Based on the measured free drug concentration in cell culture media and human serum, 1 µM VOR is above the clinical exposure achieved in patients following the oral administration of the clinical dose of 400 mg (Supplementary Table S3). Thus, RMD is capable of durable induction of HIV transcription in cells from HIV-infected patients on suppressive cART at concentrations substantially below those achieved in cancer patients following the administration of the clinically approved dose of the drug.

### RMD does not induce global activation of immune cells

**Figure 6:**
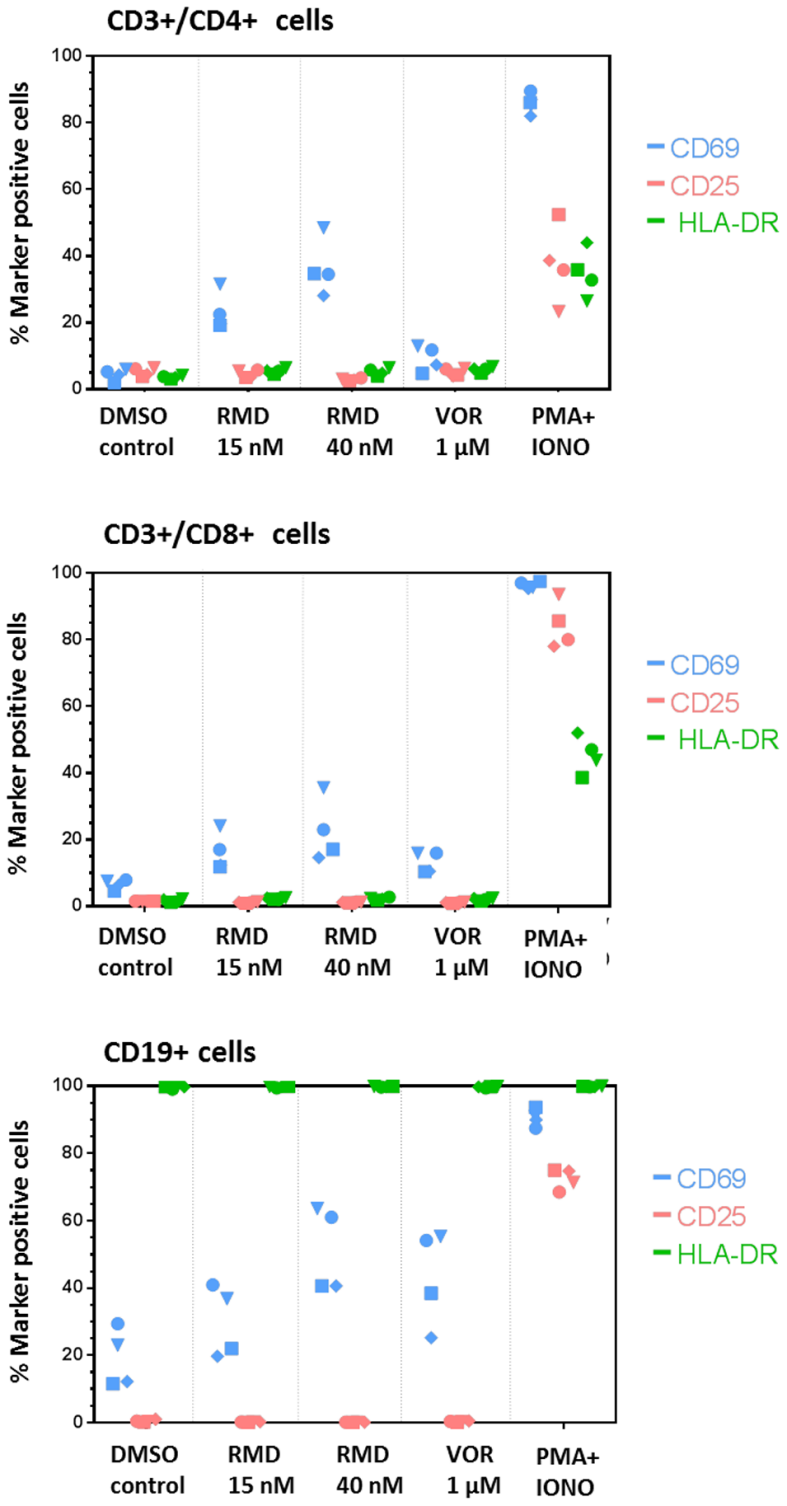
RMD does not induce global activation of immune cell subsets. PBMCs isolated from four HIV-infected patients on suppressive cART were treated with a 4-hour pulse of RMD or continuously with vehicle control (DMSO), VOR, or PMA+ionomycin and stained for surface markers 48 hours after the treatment initiation. Fractions of CD69−, CD25−, and HLA-DR-positive cells in subsets of CD4+ T cells, CD8+ T cells, and CD19+ B cells were analyzed by flow cytometry as described in Materials and Methods. Each symbol represents one donor.

HIV latency reversing agents that would be suitable for the therapeutic use in vivo should not induce non-specific immune activation. To determine if RMD may activate immune cells, PBMCs isolated from HIV-infected subjects on suppressive cART were treated with a 4-hour pulse of 15 or 40 nM RMD. The activation status of selected immune cell subsets, including CD4+ and CD8+ T cells, as well as CD19+ B cells was assessed by flow cytometry analysis of cellular activation markers relative to treatment with either 1 µM VOR or vehicle control. While RMD treatment induced dose-dependent expression of CD69 in 10% to 50% of T and B cells, it did not lead to any changes in the expression of other prominent cell activation markers such as CD25 or HLA-DR in any of the cell subsets (Fig. 6). In contrast to effects observed in the context of complete PBMC cultures, RMD treatment induced minimal increases in the fraction of CD69-positive cells in resting CD4 T cells isolated from cART-suppressed HIV-infected patients (Supplementary Fig. S2). In addition, no significant induction of IFN-α, IFN-γ, TNF- α, TGF-β, IL-2, IL-7 or other cytokines were detected in PBMC cultures from the same patients following the treatment with RMD (data not shown). Together, these data indicate that RMD effectively activates the expression of latent HIV without inducing the global activation of T or B cells.

### RMD exhibits consistent ex vivo HIV activation in longitudinal samples from the same donor

**Figure 7:**
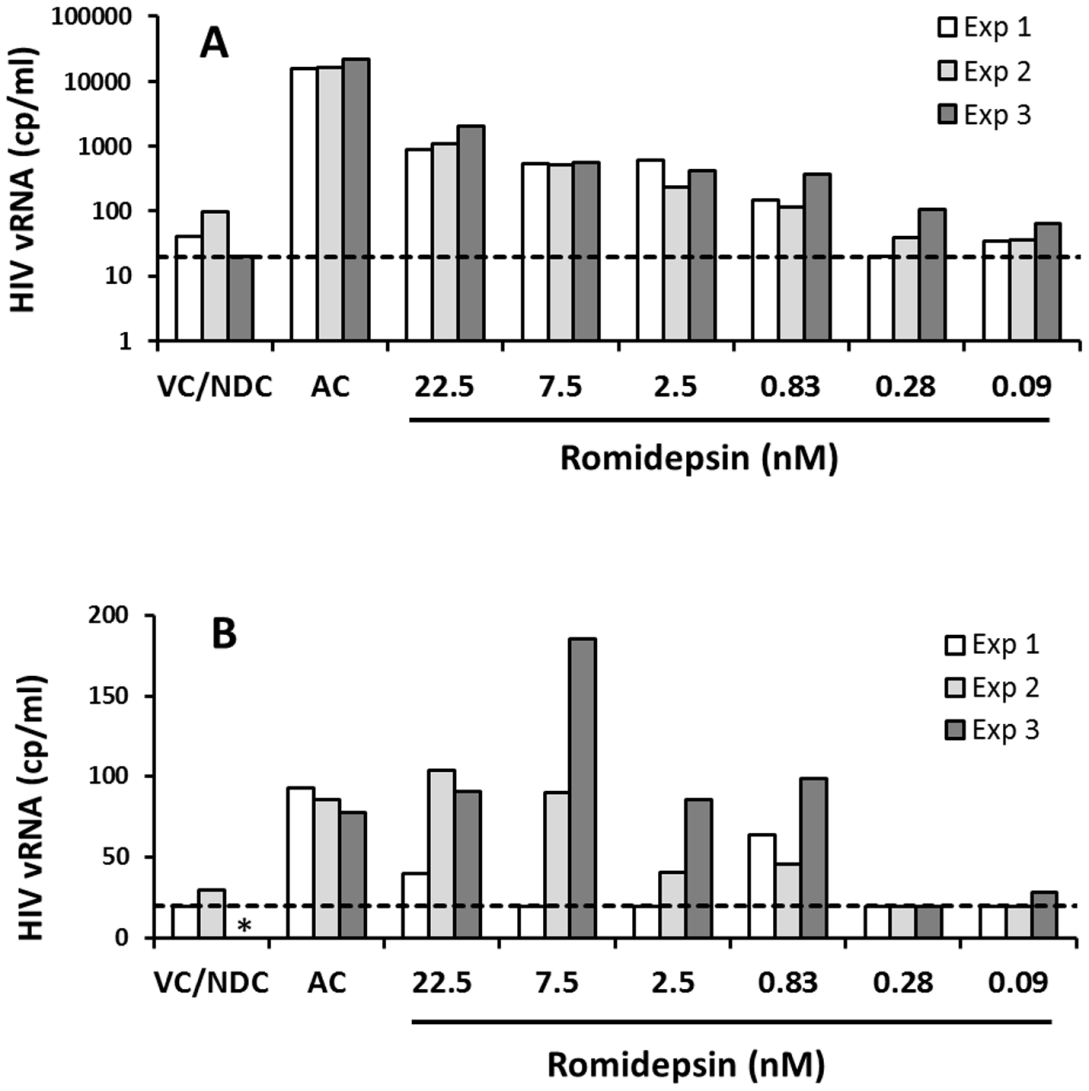
Ex vivo response to RMD in multiple longitudinal samples from the same donors. Resting CD4 T cells isolated from 2 cART-suppressed HIV-infected patients were treated continuously with RMD or with anti-CD3/CD28 antibodies (AC, activation control) for 7 days. HIV RNA in cell culture supernatants was quantified by COBAS on day 7. Data for a high-responding (**A**) and a low-responding donor (**B**) to RMD is shown; each donor was tested at 3 different time points separated by at least 2 weeks (Exp 1–3). VC/NDC, vehicle control/no-drug control. Asterisk (*) indicates no value due to COBAS analysis failure. Dashed lines indicate the limit of HIV quantification by COBAS (20 copies/ml). Data for each individual donor and condition including results of the statistical analysis are summarized in Supplementary Table S4.

Recent results showed inter-patient variation in ex vivo HIV activation by VOR [25]. Thus, it was important to determine whether HIV reactivation by RMD was consistent in samples collected longitudinally from the same patient over time. We measured HIV RNA levels following RMD treatment of resting CD4 T cells from three sequential blood samples collected several weeks apart from the same two HIV-infected patients with suppressed plasma viral load. One of the tested subjects showed robust and reproducible dose-dependent HIV RNA increase in culture supernatants after 7 days of RMD treatment in all three longitudinal samples (Fig. 7; high-responding patient). Although the other subject exhibited weaker HIV activation following treatment with RMD, a concentration-dependent effect on viral expression was observed in 2 of 3 longitudinal samples (Fig. 7; low-responding patient). These results show that RMD is capable of eliciting reproducible reactivation of latent HIV ex vivo in samples collected longitudinally from the same patient.

### Sequencing analysis of HIV induced by RMD

**Figure 8:**
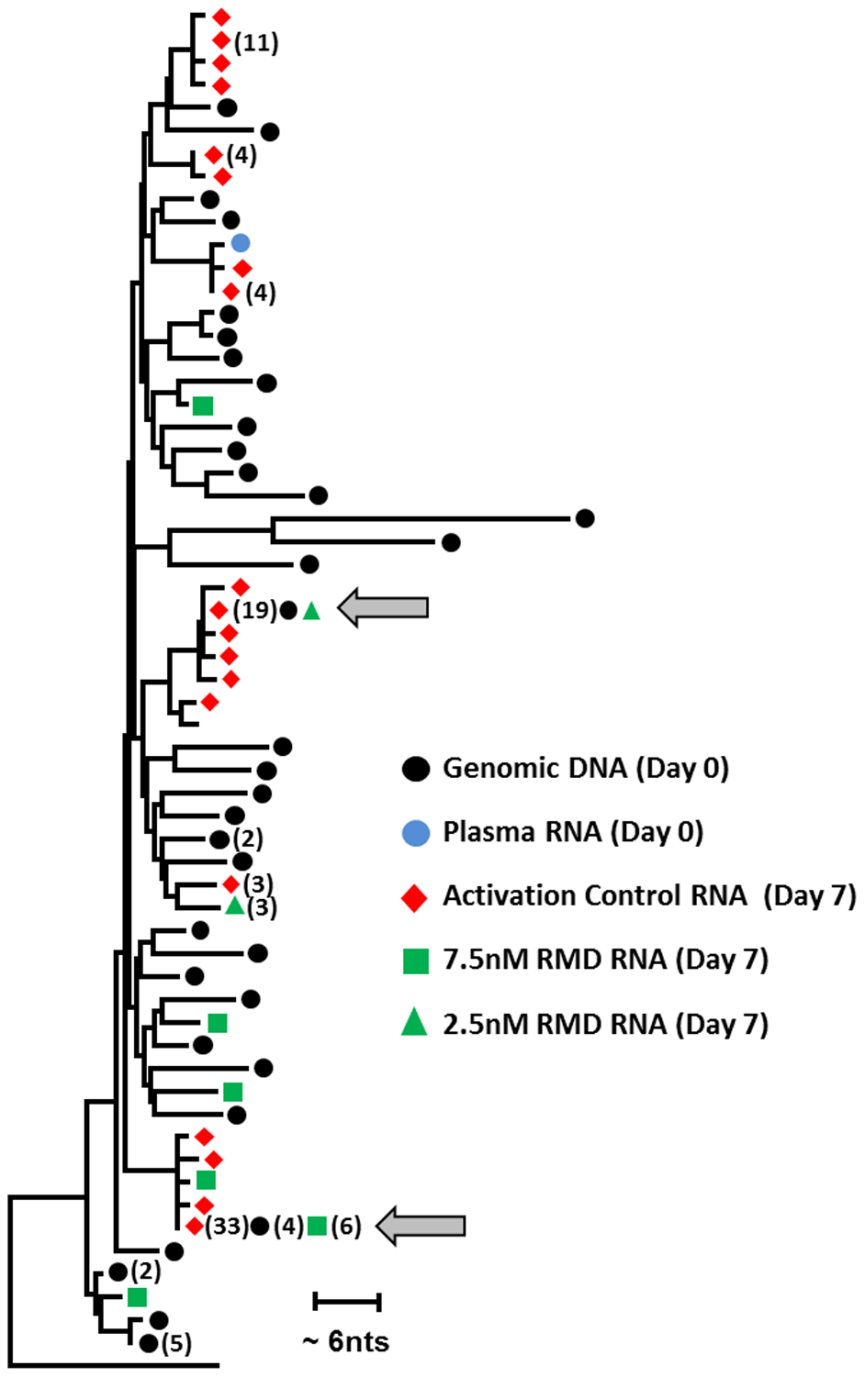
Phylogenetic analysis of HIV sequences expressed ex vivo following the latency reversal. Resting CD4 T cells isolated from a cART-suppressed HIV-infected patient (high-responding patient from Fig. 7) were treated continuously with RMD or activation control (anti-CD3/CD28 antibodies). Single-genome sequencing was used to analyze patient HIV proviral DNA and plasma RNA at the initiation of treatment (day 0), together with the ex vivo induced HIV RNA in cell culture supernatants at the end of treatment (day 7). Total of 43 sequences were recovered for proviral DNA. Eighty-eight, 11, and 4 sequences of HIV RNA were collected in culture supernatants following the treatment with activation control, 7.5 nM RMD, and 2.5 nM RMD, respectively. Grey arrows indicate examples of full concordance between the sequence of proviral DNA and viral RNA induced either by RMD or activation control. No HIV RNA sequences were recovered in supernatants from cultures treated with control vehicle or 2 µM SAHA. Identified HIV DNA and RNA sequences were aligned and the phylogenetic tree was constructed using Clustal W and MEAG5.

We used a single-genome sequencing (SGS) [34]–[36] approach to analyze sequences of HIV *gag-pol* RNA in cell culture supernatants of resting CD4 T cells from the high-responding patient (depicted in Fig. 7) following the latency reversal by RMD and compared those with sequences of HIV RNA induced by the stimulation of CD3/CD28. In parallel, we assessed the sequence diversity of HIV proviruses integrated in genomic DNA of resting CD4 T cells from the same culture. The SGS analysis confirmed the presence of multiple HIV RNA sequences in the supernatants of resting CD4 T-cell cultures following a 7-day treatment with RMD or anti-CD3/CD28 antibodies. Importantly, several proviral DNA sequences were identified that matched some of the RMD-induced HIV RNA sequences in culture supernatants (Fig. 8). However, many proviruses did not have matching RNA sequences induced in the cell culture supernatants. This is consistent with previous observations that a large proportion of integrated proviruses are refractory to the activation by latency reversal agents [37]. Notably, a few instances were found in which identical HIV RNA sequences were induced by both RMD and anti-CD3/CD28 antibodies, but many HIV RNA sequences were also detected that are unique for each treatment. This is likely, at least in part, due to different mechanisms by which the two stimuli activate latent HIV. Although more extensive sequence analyses of samples from a larger set of HIV-infected patients on suppressive cART are needed to characterize proviruses that can be specifically activated by RMD, these initial results further confirm that RMD treatment activates a subset of latent HIV proviruses in resting CD4 T cells.

## Discussion

In this report, we show that the HDACi RMD acts as a potent activator of latent HIV with effects observed in resting and memory CD4 T cells either infected in vitro or isolated from HIV-infected patients on cART with suppressed viremia. RMD was a more potent inducer of HIV expression compared to VOR and PNB, two HDACi that are being evaluated clinically in HIV-infected patients on cART. The greater potency of RMD compared with VOR to induce HIV expression was observed in multiple patient samples and at multiple time points in both total memory and resting CD4 T cells. RMD also exhibited a greater magnitude and longer persistence of HIV RNA expression relative to VOR, and was capable of inducing the release of HIV virions from infected cells. The lasting effect of RMD compared to other HDACi may be related to its unique intracellular pharmacology and interaction with HDAC enzymes. RMD acts as an intracellular prodrug that undergoes reduction of its intramolecular disulfide bond upon entering cells [38]. The released free sulfhydryl groups tightly interact with the Zn^2+^ ion in the active site of various target HDAC isoforms, a mechanism of inhibition that does not apply to VOR or PNB.

In addition, RMD activated latent HIV ex vivo in cells from HIV-infected patients on cART at concentrations that are lower than those achieved in cancer patients receiving the clinically approved dose of 14 mg/m^2^. In patients with either hematologic malignancies or solid tumors, RMD showed an acceptable short-term safety profile and was not associated with severe toxicities [33]. The most common adverse reactions related to once-weekly dosing of RMD were thrombocytopenia, neutropenia, lymphopenia, nausea, and fatigue [33].

In our study, we observed reproducible ex vivo activation of HIV by RMD at concentrations corresponding to systemic clinical exposures expected after dosing at 2–5 mg/m^2^. Given these results and the established clinical safety profile of RMD, clinical testing is warranted to assess whether RMD can activate latent HIV and potentially reduce the size of the latent reservoir in HIV-infected patients on suppressive cART.

Recently, results from two clinical studies of single and multiple doses of VOR administered to HIV-infected patients on cART have been reported. The first study demonstrated a mean 4.8-fold increase in cell-associated HIV RNA levels in resting CD4 T cells following the administration of a single 400 mg dose of VOR to 8 subjects [25]. Another study examined once-daily dosing of VOR for 14 consecutive days and reported an approximate 3-fold mean increase in cell-associated HIV RNA levels in a cohort of 20 patients [39]. However, increases in the level of plasma viremia were not observed in either study. This finding could be consistent with our observation that VOR does not cause release of virions as measured by extracellular HIV RNA. In addition to VOR, multiple doses of PNB have been shown to induce intracellular HIV transcription and low-level viremia in peripheral blood of HIV-positive patients on suppressive cART [40].

Although monitoring the induction of HIV RNA expression in resting CD4 T cells from virally suppressed subjects treated with VOR was shown to be feasible, determining whether such treatments with HDACi can affect the size of the latent virus reservoir remains a challenge. There was no apparent effect on total proviral DNA in peripheral CD4 T cells in the multiple-dose (14-day) trial of VOR [39]. This outcome may be explained in part due to the presence of a high background of defective proviruses that cannot be activated and thus would not undergo depletion from viral cytopathic effect or immune-based clearance. In addition, Ho et al. recently showed that a large fraction of intact proviruses are refractory to latency reversal [37]. Therefore, in future clinical trials of HIV latency-reversing agents, it will be critical to use methods that will allow more direct assessment of the changes in the size of the inducible HIV reservoir following its pharmacological activation.

Induction of HIV expression from latent reservoirs is being pursued as a component of the reservoir eradication strategy that may ultimately lead to a prolonged drug-free remission or even cure [11], [12]. It should be noted that RMD activates latent HIV to a lesser degree than the mitogenic T-cell activators PMA+ionomycin or anti-CD3/CD28 antibodies; therefore, additional significant efforts would likely have to be devoted to improving the HIV activation effects of RMD. As already suggested, this may potentially be achieved by combining HDACi with other latency-reversing agents that work through complementary mechanisms. For example, synergistic effects on HIV expression have been documented in vitro between VOR and the PKC activator prostratin [41]. Other classes of agents shown to activate latent HIV in vitro and/or ex vivo include histone methyltransferase inhibitors [42], [43], as well as other small molecules such as disulfiram [44], [45]. Currently, however, no specific combination of agents suitable for testing in patients has emerged. Therefore, in future studies, the latency reversing agents should be considered for testing in combination with RMD to identify potential synergies for HIV activation. Concomitantly, any identified combinations of synergistic agents would have to undergo rigorous safety evaluations as the combined safety profiles of compounds independently modulating gene expression cannot be easily deduced.

At this early discovery stage of novel agents that activate latent HIV, it is also important to realize that the fate of cells expressing pharmacologically reactivated proviruses is not fully understood. While acute HIV infection of CD4 T cells is associated with cell death via both direct and bystander mechanisms, it is unknown whether reactivation of latent HIV with RMD or other latency-reversing agents would lead to virus-induced cell death. Recent data generated in an in vitro model of HIV latency has shown that HIV activation with VOR does not lead to cell death [46]. However, this result could be at least in part attributed to the nature of the latency model that relied on the ectopic expression of the anti-apoptotic factor Bcl2 to prolong the viability of the latently infected cells and/or to other unrecognized differences between in vitro models and the in vivo state. Addressing the fate of infected CD4 T cells after virus reactivation with RMD is a high priority of ongoing studies.

If reactivation of latent virus does not lead to cell death, additional interventions would be needed to eliminate cells expressing viral proteins. Strategies may include agents that selectively enhance apoptosis in infected cells following the latency reversal [47]. Other approaches under consideration include antibody-based therapies to recruit immune effector cells such as natural killer cells via antibody-dependent cell-mediated cytotoxicity [48], a strategy that has been used clinically for the treatment of various malignancies [49]. One potential advantage of the antibody-mediated clearance of virally infected cells is the high degree of selectivity of monoclonal antibodies for cells expressing the HIV envelope antigen. However, it is unclear whether the pharmacological activation of latent HIV will induce sufficient levels of gp120/gp41 expression on the surface of the treated cells to trigger an antibody-mediated effector mechanism resulting in cell lysis. Although we and others have detected intracellular HIV p24 capsid protein following treatment with HDACi using in vitro HIV latency models, little is known about levels of viral protein expression that can be induced by HDACi in latently infected resting CD4 T cells from cART-treated patients. One of the major challenges in addressing this question is the very low frequency of T cells harboring latent HIV provirus in virally suppressed patients [37]. New approaches to selectively enrich the fraction of latently infected cells isolated from patients are being explored [50] and may ultimately enable more in-depth characterization of RMD and other latency-reversing agents.

It should be noted that the treatment with HDACi could modify immune functions and responses (reviewed in [51], [52]). Various HDACi have been shown to inhibit innate immunity responses and cytokine production [53], [54] as well as immune cell trafficking and functions [55]–[57]. While the clinical doses of RMD approved for oncology applications can diminish responses of immune effector cells in lymphoma patients [58], our data support the exploration of lower doses of RMD for the activation of latent viral reservoirs in HIV-infected patients. Such modified dosing regimens could reduce or eliminate the perturbations of immune functions potentially associated with RMD treatment. In addition, treatment with agents that stimulate innate immunity such as TLR7/8 agonists could mitigate the suppressive effects of RMD on the activity of immune effector cells [58]. If RMD use eventually requires combination with immunostimulatory strategies to enhance antiviral immune effector functions and the clearance of latent HIV reservoirs, optimized temporal separation in the administration of RMD and immune stimulators could further minimize the potential of RMD-mediated interference with immune responses.

In summary, this report shows that RMD is a more potent and robust inducer of HIV expression in latently infected cells compared with other HDACi in clinical testing. This profile warrants the assessment of RMD in virally suppressed HIV-infected patients.

## Materials and Methods

### Ethics statement

HIV-infected patients were enrolled into the study at the Quest Clinical Research (QCR; San Francisco, CA) and the University of Pittsburgh Medical Center (UPMC; Pittsburgh, PA). The QCR and the UPMC part of the study were approved by the Western Institutional Review Board and the University of Pittsburgh Institutional Review Board, respectively. In both cases, written, informed consent was obtained from the patients prior to any study procedures.

### HDAC inhibitors

Romidepsin was obtained from a US-based pharmacy as a marketed commercial formulation of the drug. Vorinostat, panobinostat, givinostat, mocetinostat and SB939 were obtained from Selleck Chemicals (Houston, TX). Phorbol 12-myristate 13-acetate (PMA) and ionomycin were obtained from Sigma Aldrich (St. Louis, MO). All compounds were dissolved in DMSO. Effects of HDAC inhibitors in all assays and models used in this study have been compared to vehicle (i.e. DMSO) treated controls.

### Construction of HIV expressing a luciferase reporter gene

Plasmid pKS13 is a NL4-3-based vector in which the *vpr* and *env* genes were inactivated by inserting a ‘T’ base at the AflII site and two bases (TT) at the NdeI site, respectively, causing frame shifts in both open reading frames. A codon-optimized firefly luciferase gene was introduced in place of the *nef* gene by replacing the BamHI and NcoI fragment, yielding the plasmid pKS13. NL4-3-Luc virus was generated by co-transfection of HEK-293T cells with pKS13 and a plasmid containing the HIV-1 *env* gene using Lipofectamine 2000 (Life Technologies, Grand Island, NY).

### In vitro model of HIV latency

Total peripheral blood mononuclear cells (PBMCs) were obtained from healthy HIV-negative donors by leukapheresis (AllCells, Inc, Emeryville, CA). Naive CD4+ T cells were purified by negative selection using EasySep magnetic beads (StemCells, Inc, Vancouver, Canada) and cultured in RPMI with 10% fetal bovine serum (FBS), penicillin/streptomycin, 1% nonessential amino acids (Life Technologies, Carlsbad, CA), 1% sodium pyruvate (Life Technologies) and 495 nM beta-mercaptoethanol (Sigma Aldrich, St. Louis, MO) in a 37°C, 5% CO_2_ incubator. Purified naive CD4+ T cells were activated by incubation with anti-CD3/CD28 magnetic Dynabeads (1 bead: 2 cells ratio, Life Technologies), 1 µg/ml anti-IL-4 (R&D Systems, Minneapolis, MN), 2 µg/ml anti-IL-12p70 antibodies (R&D Systems), and 10 ng/ml TGF-β (R&D Systems) for 3 days [29], [59]. Following the removal of anti-CD3/CD28 beads and antibodies, cells were maintained in 30 U/ml IL-2 (Life Technologies) for 2 days. Cells were then infected with NL4.3-Luc in the presence of 50 µg/ml DEAE for 3 hours. Cells were maintained in the continued presence of 30 U/ml IL-2 throughout the infection and subsequent rest period with culture medium with fresh IL-2 replaced every 2–3 days. Seven days post-infection, 20 µl of latently infected cells were dispensed into 384 well plates using a MicroFlo dispenser (Biotek Insturments, VT) at 10,000 cells/well containing 100 nl of compound solutions delivered by the Echo acoustic-based liquid dispenser (Labcyte, Sunnyvale, CA). After a 48-hour incubation, 16 µl/well BriteGlo (Promega, Madison, WI) was added and luminescence measured using the Envision plate reader (Perkin Elmer, Waltham, MA). Compound-associated cytotoxicity was determined in latently infected cells in parallel with the virus activation assay. Cells were incubated with compounds for 48 hours at 37°C, and cell viability was determined using Cell Titer Glo reagent (Promega).

### Flow cytometry analysis of p24 expression

Seven days post-infection, cells infected with NL4-3-Luc virus were stimulated for 48 hours with test compounds. Cells were stained with fixable viability dye v450 (eBioscience, San Diego, CA) in phosphate buffered saline (PBS) +2% FBS for 30 minutes at 4°C. Cells were fixed with IC Fixation Buffer (eBioscience) for 20 minutes at room temperature in the dark followed by treatment with a permeabilization buffer (eBioscience) for 30 minutes at 4°C. Intracellular p24 was stained with anti-p24 antibody clone KC57-RD1 (Beckman Coulter, Fullerton, CA), then washed and resuspended in PBS +2% FBS. The cells were analyzed on a LSR Fortessa (BD Biosciences, San Jose, CA) and collected data were processed using a FlowJo Analysis Software (TreeStar, Ashland, OR).

### HDAC inhibition assays

Determination of HDACi potency against individual HDAC enzymes was performed at Reaction Biology Corporation (Malvern, PA). In brief, human HDAC-1 to -11 enzymes were incubated with serial dilutions of HDACi and fluorogenic peptide substrates (50 µM) in a 96-well format assay. Concentrations of HDACi reducing the control activity of tested enzymes by 50% (IC_50_) were determined by a five-parameter curve fit of collected fluorescent signals. Fluorogenic peptide derived from p53 protein (amino acid residues 379–382; RHKK_Ac_) was used as a substrate for HDAC enzymes 1, 2, 3, 6, 10, and 11. A different fluorogenic peptide from p53 (amino acid residues 379–382, RHK_AC_K_AC_) was used as a substrate for HDAC-8. Fluorogenic peptide Boc-Lys(trifluoroacetyl)-AMC was used as a substrate for HDAC enzymes 4, 5, 7, and 9.

### Ex vivo activation of HIV transcription

HIV-infected patients participating in the study were selected based on sustained plasma viral load suppression (<50 copies/ml for >12 months), CD4 counts (>350 cells/µL), and absence of co-infection with hepatitis B or C virus (Supplementary Table S1). Clinical laboratory results were reconfirmed 2 weeks before leukapheresis or blood draw. Leukapheresis was conducted for 3–4 hours, and samples were processed within 2 hours after collection. The leukapheresis product was diluted 1∶1 with PBS and layered over Ficoll for isolation of PBMCs. PBMCs were treated with red blood cell lysis buffer (eBioscience) and rested overnight (10 million cells/ml) in tissue culture medium (RPMI 1640 supplemented with 10% FBS and PenStrep) before the isolation of memory CD4 T cells according to the manufacturer's recommendation (EasySep Human Memory CD4 T cell Enrichment Kit). Resting CD4 T cells were isolated by first enriching total CD4 T cells (StemCell Technologies) and subsequently depleting HLA-DR-, CD25-, and CD69-positive cells via negative selection (Miltenyi Biotec, Auburn, CA). Flow cytometry was used to assess the purity of both T-cell subsets (>98%). Resting CD4 T cells for the analysis of continuous RMD exposure and longitudinal responses to RMD in the same donors were purified from fresh blood using the same protocol.

To assess HIV activation by HDACi, up to 5 million CD4 T cells were plated in 24-well plates in 2.5 ml of media, supplemented with antiretrovirals (ARVs) (100 nM elvitegravir and 100–300 nM efavirenz) for the entire duration of culture incubation. HDACi were added at specified concentrations on day 0 and cells or culture supernatants were harvested for the analysis of HIV RNA at the indicated time points (6–48 hours or 6–7 days, respectively). For HDACi pulse treatment, cultures were washed twice using ARV-containing media at 4 or 24 hours after addition of HDACi and incubated with ARVs until harvest. To measure HIV RNA levels, 1 ml of culture supernatant was analyzed by a robotic COBAS AmpliPrep/TaqMan system (Roche Diagnostics, Indianapolis, IN), which extracts total nucleic acid and quantifies HIV RNA in copies per milliliter using the HIV-1 Test, v2.0 kit (Roche Diagnostics). For the measurement of cell-associated HIV RNA levels, cells were washed with PBS, counted, and lysed (Qiagen RLT buffer, 400 µl for every 2 million cells; Qiagen, Venlo, Netherlands). Lysates were filtered through a Qiashredder (Qiagen). Total RNA was prepared from the lysates (400 µl) using a robotic system (QIAsymphony, Qiagen) that incorporates a DNase I digestion step to eliminate cellular DNA. The resulting total RNA was eluted with 200 µl of buffer, diluted to 1 ml with nuclease-free water, and analyzed by COBAS using the HIV-1 Test v2.0 kit. Preparations of both cell-associated RNA and supernatant total nucleic acids were tested for potential contamination with HIV DNA and/or host DNA by performing the PCR amplification in the presence and absence of reverse transcriptase and by a detection of GAPDH-encoding host DNA sequence. These methods confirmed that there was no contaminating HIV DNA in either the intracellular RNA or supernatant total nucleic acid preparations (Supplementary Figs. S3 and S4).

### Cell activation analysis by flow cytometry

For cell activation analysis, either complete PBMC cultures or resting CD4+ T cells isolated from HIV-infected cART-suppressed patients were incubated with the tested agents under specified conditions and were stained with relevant antibodies. All antibodies were purchased from BD Biosciences and included Alexa Fluor 700-labeled antibody to CD4, APC-H7-labeled antibody to CD8, PE-Cy7 -labeled antibody to CD19, PE-labeled antibody to CD69, APC-labeled antibody to CD25, and V450-labeled antibody to HLA-DR. Live cells were gated by forward and side scatter and exclusion of the dead cells by Live/Dead Fixable Aqua Stain (Invitrogen). Marker staining was assessed by flow cytometry analysis on a LSR Fortessa with data processing using FlowJo software.

### Cell-associated HDAC enzymatic assay

Resting CD4 T cells obtained from HIV-infected patients on suppressive cART were incubated with serial dilutions of HDACi in 96-well plates at 10,000 cells/well for indicated time and then lysed by repeated freezing and thawing. Total cellular HDAC activity was measured using the HDAC-Glo I/II assay kit (Promega) according to the manufacturer's protocol.

### Phylogenetic analysis of HIV DNA and RNA during ex vivo latency reversal

Single-genome sequencing of a portion of HIV-1 *gag-pro-pol* amplified from cell culture supernatants or cellular DNA extracts was performed as previously described [34]–[36]. Sequences were aligned using ClustalW and neighbor-joining phylogenetic analysis was performed using MEGA5. Trees were rooted on the subtype B consensus sequence (www.hiv.lanl.gov).

### Experimental and statistical analyses

HIV RNA-induction experiments were conducted using 5 to 6 replicates for every experimental condition, including vehicle-treated controls. Fold induction in HIV RNA levels was calculated as a ratio of mean signals from compound-treated and vehicle (DMSO)-treated control samples from the same donor under identical conditions. Student's t-test (one-tailed distribution, two-sample equal variance) was used to assess statistical significance of the difference between the detected HIV RNA copies in vehicle control- and HDACi-treated samples or between RMD- and VOR-treated samples. Time course experiments testing the latency reversal agents had matching vehicle-treated controls for each time point. For experiments using blood-derived resting CD4 T cells, culture supernatants from 3 replicate wells were combined and analyzed using the COBAS system. Primary results in absolute HIV RNA copy numbers from all experiments presented in this study are provided in Supplementary material (Supplementary Table S4).

## Supporting Information

Figure S1 **Effect of RMD and VOR on the viability of primary memory CD4 cells.** Memory CD4 cells were isolated from HIV-infected patients and treated with RMD or VOR for 4 or 24 hours, respectively. Cell viability was determined using a Cell TiterGlo reagent 6 days after the initiation of treatment and is expressed as a percentage of cell viability relative to control vehicle-treated memory CD4 cells from the same donors. The data represent mean +/− S.D. from three independent donors.
(TIF)

Figure S2 **Effects of RMD and VOR on the expression of activation markers in primary resting CD4+ T cells, compared to the effect of PMA plus ionomycin stimulation.** Cells were treated with a 4-hour pulse of the indicated concentrations of RMD or continuously with either VOR or PMA+ ionomycin (positive control), followed by staining 48 hours later. The surface expression of CD25 and CD69 in viable CD4 cells was analyzed by flow cytometry. Data are mean ± SD of two independent experiments performed with cells isolated from two HIV-infected patients on suppressive cART.
(TIF)

Figure S3 **Lack of HIV DNA contamination in extracted intracellular RNA samples following the treatment with DNase I.** (**A**) Two million memory CD4 T cells isolated from three HIV-infected cART-suppressed patients (Donors A–C) were treated with control (blank, bk) or romidepsin (RMD) for 48 hours, washed, lysed, and filtered through a Qiagen shredder to obtain homogenized cell lysates before additional analyses. Cell lysates were extracted using QIAsymphony, with or without DNase I digestion, before the entire sample was analyzed by COBAS for the quantification of HIV viral sequences. (**B**) Cells from identical donors were lysed, shredded, and then extracted for total RNA using QIAsymphony with DNase I digestion. Samples aliquots were analyzed by qPCR for HIV Gag and GAPDH sequences, with or without addition of reverse transcriptase (RT+ or RT−). Asterisks (*) indicate none detected. (**C**) Random lysates of vehicle-treated memory CD4 T cells from virally suppressed HIV patients (#1–8) were divided into identical duplicates and extracted for total RNA using QIAsymphony with DNase I digestion. The total RNA was then treated with additional DNase I digestion or not (yes vs. no) before quantification of HIV viral sequences by COBAS.
(TIF)

Figure S4 **Lack of HIV DNA contamination in total nucleic acid extracts from cell culture supernatants.** Memory CD4 cells isolated from four HIV-infected cART-suppressed patients (Donors A–D) were treated with no drug control (blank; bk), 5 nM romidepsin (RMD) or PMA+ ionomycin (P/I) for 6 days. Cell culture supernatants were extracted for total nucleic acid (tNA) using COBAS TNAI kit before additional analyses. (**A**) HIV Gag DNA and host GAPDH DNA were quantified in tNA by qPCR without reverse transcriptase. Asterisks (*) indicate none detected. (**B**) The same tNA samples were further incubated with or without DNase I (yes vs. no), re-extracted for tNA, and analyzed for HIV copies by COBAS HIV viral load analyzer. Hash marks (#) indicate the limit of HIV quantification (<20 copies/ml).
(TIF)

Table S1 **Demographic characteristics of HIV-infected patients participating in the study.**
(XLS)

Table S2 **HIV RNA released from resting CD4 T cells treated with RMD can be pelleted by high-speed centrifugation.** ^a^ Percentage of total nucleic acid in the sample. Resting CD4 T cells isolated from an HIV-infected patient on suppressive cART were treated with RMD for 6 days and the collected supernatants were subjected to ultracentrifugation (21,000 g×60 min). HIV DNA and RNA were quantified in pellet and supernatant using Taqman quantitative PCR.
(DOCX)

Table S3 **Systemic clinical exposures of RMD and VOR compared to concentrations used in the ex vivo experiments.** ^a^ Istodax (romidepsin) prescribing information (www.istodax.com). ^b^ Zolinza (vorinostat) prescribing information www.zolinza.com/vorinostat/zolinza).^c^ Determined by an equilibrium dialysis followed by HPLC/mass spectrometry analysis. ^d^ Ratio of free drug concentration in cell culture media and free drug concentration in serum of clinically treated patients.
(DOCX)

Table S4 **Summary of datasets from analyses of HIV RNA induction in the ex vivo primary CD4 T cell cultures isolated from virologically suppressed HIV-infected patients.** The table shows compiled primary data and statistical analyses from the quantitation of HIV RNA (copies/million cells for intracellular HIV RNA; copies/mL for supernatant HIV RNA) in various types of CD4 T cell cultures isolated from HIV-infected patients and treated with tested HDACi or vehicle control. The datasets represent results displayed in Figures 2, 3, 4, 5, and 7.
(XLS)
