# Supplementary figures and images for: Histone Deacetylase Inhibitor Romidepsin Induces HIV Expression in CD4 T Cells from Patients on Suppressive Antiretroviral Therapy at Concentrations Achieved by Clinical Dosing
Source: PLoS Pathog. 2014 Apr 10;10(4):e1004071. doi: 10.1371/journal.ppat.1004071 (PMC3983056; doi:10.1371/journal.ppat.1004071)

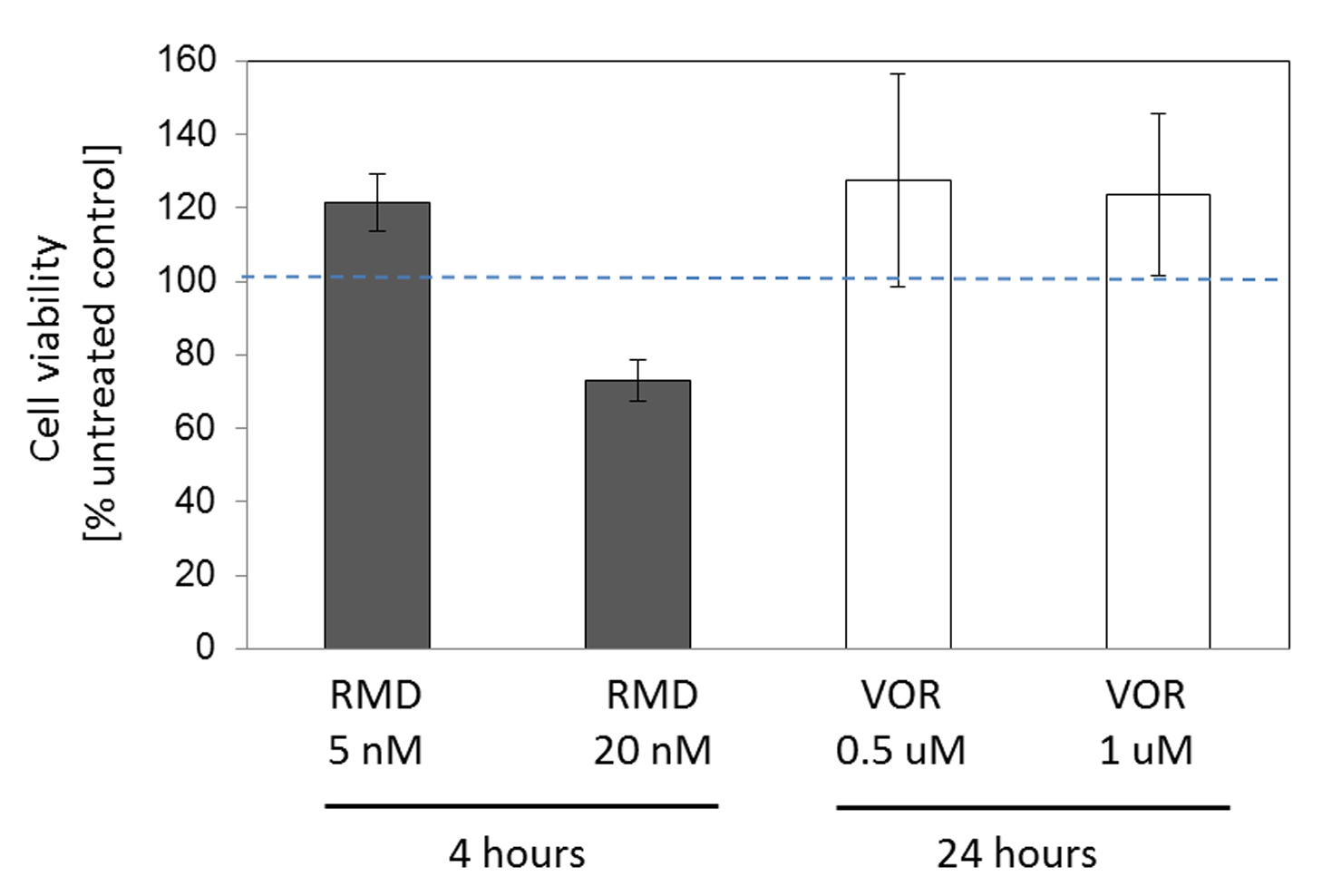

Supplement: Figure S1 — Effect of RMD and VOR on the viability of primary memory CD4 cells. Memory CD4 cells were isolated from HIV-infected patients and treated with RMD or VOR for 4 or 24 hours, respectively. Cell viability was determined using a Cell TiterGlo reagent 6 days after the initiation of treatment and is expressed as a percentage of cell viability relative to control vehicle-treated memory CD4 cells from the same donors. The data represent mean +/− S.D. from three independent donors. (TIF) [file ppat.1004071.s001.tif]

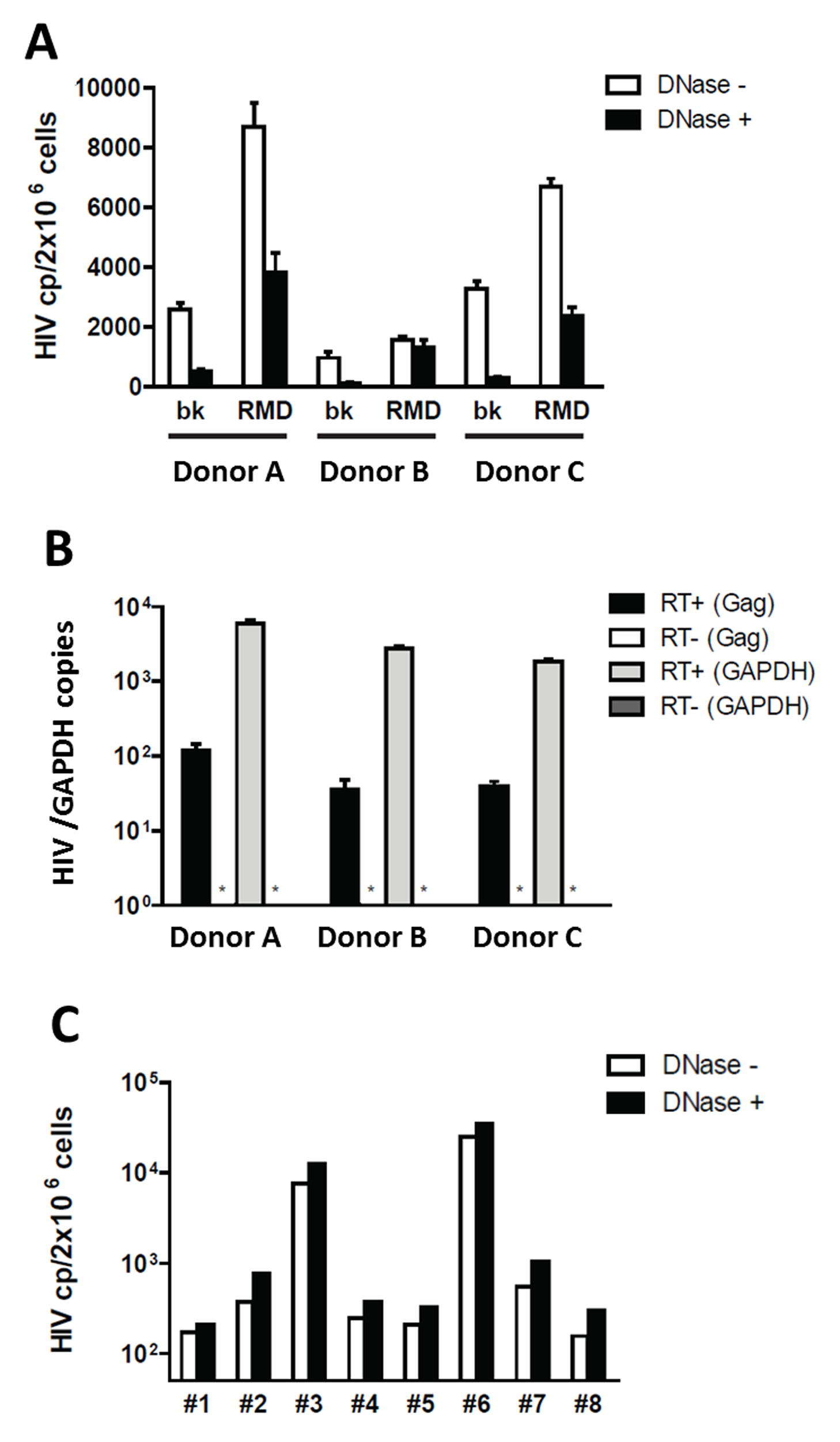

Supplement: Figure S3 — Lack of HIV DNA contamination in extracted intracellular RNA samples following the treatment with DNase I. (A) Two million memory CD4 T cells isolated from three HIV-infected cART-suppressed patients (Donors A–C) were treated with control (blank, bk) or romidepsin (RMD) for 48 hours, washed, lysed, and filtered through a Qiagen shredder to obtain homogenized cell lysates before additional analyses. Cell lysates were extracted using QIAsymphony, with or without DNase I digestion, before the entire sample was analyzed by COBAS for the quantification of HIV viral sequences. (B) Cells from identical donors were lysed, shredded, and then extracted for total RNA using QIAsymphony with DNase I digestion. Samples aliquots were analyzed by qPCR for HIV Gag and GAPDH sequences, with or without addition of reverse transcriptase (RT+ or RT−). Asterisks (*) indicate none detected. (C) Random lysates of vehicle-treated memory CD4 T cells from virally suppressed HIV patients (#1–8) were divided into identical duplicates and extracted for total RNA using QIAsymphony with DNase I digestion. The total RNA was then treated with additional DNase I digestion or not (yes vs. no) before quantification of HIV viral sequences by COBAS. (TIF) [file ppat.1004071.s003.tif]

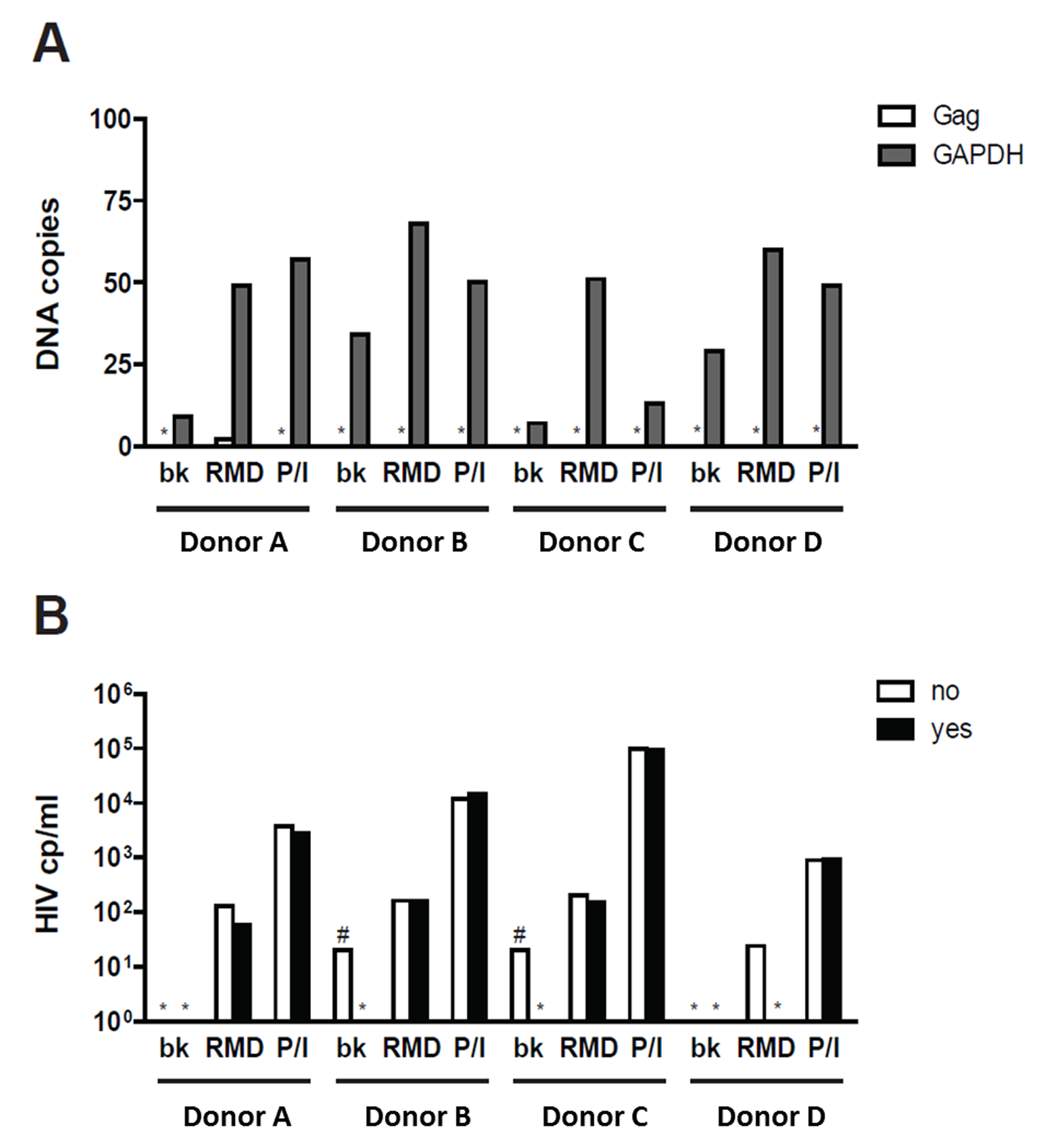

Supplement: Figure S4 — Lack of HIV DNA contamination in total nucleic acid extracts from cell culture supernatants. Memory CD4 cells isolated from four HIV-infected cART-suppressed patients (Donors A–D) were treated with no drug control (blank; bk), 5 nM romidepsin (RMD) or PMA+ ionomycin (P/I) for 6 days. Cell culture supernatants were extracted for total nucleic acid (tNA) using COBAS TNAI kit before additional analyses. (A) HIV Gag DNA and host GAPDH DNA were quantified in tNA by qPCR without reverse transcriptase. Asterisks (*) indicate none detected. (B) The same tNA samples were further incubated with or without DNase I (yes vs. no), re-extracted for tNA, and analyzed for HIV copies by COBAS HIV viral load analyzer. Hash marks (#) indicate the limit of HIV quantification (<20 copies/ml). (TIF) [file ppat.1004071.s004.tif]
