# Supplementary material for: Histone Deacetylase Inhibitor Romidepsin Induces HIV Expression in CD4 T Cells from Patients on Suppressive Antiretroviral Therapy at Concentrations Achieved by Clinical Dosing
Source: PLoS Pathog. 2014 Apr 10;10(4):e1004071. doi: 10.1371/journal.ppat.1004071 (PMC3983056; doi:10.1371/journal.ppat.1004071)
Supplement: Table S2 — HIV RNA released from resting CD4 T cells treated with RMD can be pelleted by high-speed centrifugation. a Percentage of total nucleic acid in the sample. Resting CD4 T cells isolated from an HIV-infected patient on suppressive cART were treated with RMD for 6 days and the collected supernatants were subjected to ultracentrifugation (21,000 g×60 min). HIV DNA and RNA were quantified in pellet and supernatant using Taqman quantitative PCR. (DOCX) [file ppat.1004071.s006.docx]

|  | **Supernatant** | | **Pellet** | |
| --- | --- | --- | --- | --- |
| **Treatment** | **DNA, copies (%total NA)** **^a^** | **RNA, copies (%total NA)** | **DNA, copies (%total NA)** | **RNA, copies (%total NA)** |
| Control | 0 (0) | 2 (100) | 4 (100) | 0 (0) |
| Anti-CD3/28 | 4 (100) | 0 (0) | 52 (5.3) | 930 (94.7) |
| 22.5 nM RMD | 0 | 0 | 13 (4.2) | 295 (95.8) |
| 7.5 nM RMD | 2 (25) | 7 (75) | 13 (14) | 80 (86) |
| 2.5 nM RMD | 2 (50) | 2 (50) | 13 (39.4) | 20 (60.6) |
