# Supplementary material for: Histone Deacetylase Inhibitor Romidepsin Induces HIV Expression in CD4 T Cells from Patients on Suppressive Antiretroviral Therapy at Concentrations Achieved by Clinical Dosing
Source: PLoS Pathog. 2014 Apr 10;10(4):e1004071. doi: 10.1371/journal.ppat.1004071 (PMC3983056; doi:10.1371/journal.ppat.1004071)
Supplement: Table S3 — Systemic clinical exposures of RMD and VOR compared to concentrations used in the ex vivo experiments. a Istodax (romidepsin) prescribing information (www.istodax.com). b Zolinza (vorinostat) prescribing information www.zolinza.com/vorinostat/zolinza).c Determined by an equilibrium dialysis followed by HPLC/mass spectrometry analysis. d Ratio of free drug concentration in cell culture media and free drug concentration in serum of clinically treated patients. (DOCX) [file ppat.1004071.s007.docx]

|  | **RMD** | **VOR** |
| --- | --- | --- |
| **Clinical systemic exposure** |  |  |
| Dose | 14 mg/m^2^ i.v. | 400 mg p.o. |
| Peak serum concentration (C_max_) | 377 ng/mL ^a^ (698 nM) | 1,200 nM ^b^ |
| Human serum binding (free fraction) | 8% ^c^ | 30% ^c^ |
| Free drug concentration | 56 nM | 360 nM |
| **Ex vivo cell culture exposure** |  |  |
| Concentration | 40 nM | 1,000 nM |
| Cell culture medium protein binding  (free fraction) | 55% ^c^ | 68% ^c^ |
| Free drug concentration | 22 nM | 680 nM |
| **Fraction of clinical exposure^d^** | **39%** | **189%** |
